# An Adenosylcobalamin Specific Whole‐Cell Biosensor

**DOI:** 10.1002/adhm.202300835

**Published:** 2023-05-01

**Authors:** Juan José Quispe Haro, Seraphine V. Wegner

**Affiliations:** ^1^ Institute of Physiological Chemistry and Pathobiochemistry University of Münster Waldeyerstrasse 15 48149 Münster Germany

**Keywords:** adenosylcobalamin, agglutination assays, biosensors, CarH, vitamin B12

## Abstract

Vitamin B12 (cobalamin) is essential for human health and its deficiency results in anemia and neurological damage. Vitamin B12 exists in different forms with various bioactivity but most sensors are unable to discriminate between them. Here, a whole‐cell agglutination assay that is specific for adenosylcobalamin (AboB12), which is one of two bioactive forms, is reported. This biosensor consists of *Escherichia coli* that express the AdoB12 specific binding domain of CarH at their surface. In the presence of AdoB12, CarH forms tetramers, which leads to specific bacterial cell–cell adhesions and agglutination. These CarH tetramers disassemble upon green light illumination such that reversion of the bacterial aggregation can serve as internal quality control. The agglutination assay has a detection limit of 500 nм AdoB12, works in protein‐poor biofluids such as urine, and has high specificity to AdoB12 over other forms of vitamin B12 as also demonstrated with commercially available supplements. This work is a proof of concept for a cheap and easy‐to‐readout AdoB12 sensor that can be implemented at the point‐of‐care to monitor high‐dose vitamin B12 supplementation.

## Graphical abstract

Adenosylcobalamin (AdoB12) is a bioactive form of vitamin B12 and essential for human health. By using the AdoB12 binding domain of CarH, an agglutination based whole‐cell biosensor that works in urine with a detection limit of 500 nм with high specificity toward AdoB12 over other vitamin B12 derivatives, is designed.

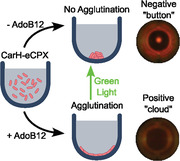

## 1 Introduction

Vitamin B12 refers to a family of organometallic molecules called cobalamins, which are essential cofactors for enzymes involved in DNA synthesis as well as lipid and amino acid metabolism. Consequently, it is essential to produce red blood cells, maintain the nervous system, and play important roles during development. Unfortunately, vitamin B12 deficiency is quite common due to nutritional imbalance, malabsorption disorders, as well as genetic defects in the uptake, transport, and intracellular processing of vitamin B12.^[^ ^1^ ^]^ This deficiency has severe consequences ranging from pernicious anemia,^[^ ^2^, ^3^, ^4^ ^]^ neurological disorders associated with irreversible demyelination of nerve cells,^[^ ^5^, ^6^ ^]^ and pregnancy complications.^[^ ^7^ ^]^ The most common treatment for these patients is the oral or parenteral administration of mega doses of up to 1000 µg daily compared to the regular required 1–2 µg per day.^[^ ^3^, ^8^, ^9^ ^]^ Different vitamin B12 derivatives all consist of a cobalt tetraazamacrocyclic complex, the cobalamin (Cbl) core, and vary in their axial ligands. Cyanocobalamin (CNB12) and hydroxycobalamin (OHB12) are often used in food supplements and are not biologically active. These forms are enzymatically converted into bioactive methylcobalamin (MeB12) and adenosylcobalamin (AdoB12), which have distinct metabolic fates and functions.^[^ ^10^ ^]^ Individuals with hereditary vitamin B12 deficiency are unable to transport or produce MeB12 and/or AdoB12 depending on the specific gene defect and require individually dosed therapy with appropriate vitamin B12 derivatives.^[^ ^11^ ^]^ Taken together, monitoring B12 levels in different body fluids is of interest in detecting deficiencies and monitoring/adjusting high‐dose therapy.

There is a large variety of methods to measure vitamin B12 that rely on detecting either vitamin B12 directly or through metabolites of vitamin B12‐dependent enzymes. These methods range from standard approaches based on liquid chromatography^[^ ^12^ ^]^ and atomic absorption spectroscopy^[^ ^13^ ^]^ to more specialized techniques including chemiluminescence,^[^ ^14^ ^]^ ELISA,^[^ ^15^ ^]^ surface plasmon resonance,^[^ ^16^ ^]^ carbon fiber electrodes,^[^ ^17^ ^]^ and nanoparticles sensors.^[^ ^18^ ^]^ Whole‐cell biosensors for vitamin B12 build on AdoB12‐responsive riboswitches^[^ ^19^, ^20^ ^]^ and vitamin B12 binding domains,^[^ ^21^ ^]^ with fluorescence outputs as well as amperometric microbial biosensors.^[^ ^22^ ^]^ Each of these methods has different limitations such as the need for large and expensive equipment, complicated reagents, long assay times, as well as low tolerance to complex matrices and poor limits of detection. Most importantly, these sensors cannot differentiate between the different bioactive and non‐bioactive forms of vitamin B12, despite their distinct roles.

Whole‐cell biosensors have great potential for affordable and point‐of‐care diagnostics. These sensors build on diverse natural sensing modules with high sensitivity and specificity for biomarkers in complex environments and combine these with detectable outputs.^[^ ^23^, ^24^ ^]^ The visual change resulting from cell agglutination is a particularly easy readout.^[^ ^25^, ^26^ ^]^ In these agglutination assays, cells clump together through the multivalent binding of an analyte to receptors expressed on the surfaces of bacterial or yeast cells, which is visible by the eye. Whole‐cell agglutination sensors have been developed for analytes with known nanobodies or to detect antibodies against known antigens.^[^ ^24^, ^25^, ^26^ ^]^

Here, we report an agglutination assay that specifically responds to AdoB12 and not to the other forms of vitamin B12. For this purpose, we suggested CarH from *Thermus thermophiles* as an appropriate sensory domain for AdoB12. CarH is a transcription factor that binds to DNA to regulate the synthesis of carotenoids in bacteria in response to light.^[^ ^27^ ^]^ At the molecular level, CarH is a monomer in its apoform and upon AdoB12 binding forms a tetramer. This tetramer is stable in the dark but under blue/green light illumination, there is a ligand exchange reaction at the AdoB12, which leads to the disassembly of the CarH tetramer into its monomers.^[^ ^28^ ^]^ CarH is an extremely versatile light‐responsive protein, which besides its applications in optogenetics to control receptor activity^[^ ^29^ ^]^ and gene expression,^[^ ^30^, ^31^ ^]^ has also been employed to generate light‐responsive hydrogels^[^ ^32^ ^]^ and protein micro‐patterning.^[^ ^33^, ^34^ ^]^ Moreover, when it is expressed as a surface receptor, it is able to mediate light‐responsive cell–cell adhesions in mammalian cells,^[^ ^35^ ^]^ which makes it a suitable candidate to also mediate bacterial cell–cell adhesions.

## 2 Results and Discussion

### 2.1 Design of the AdoB12 Sensor

**Figure 1:**
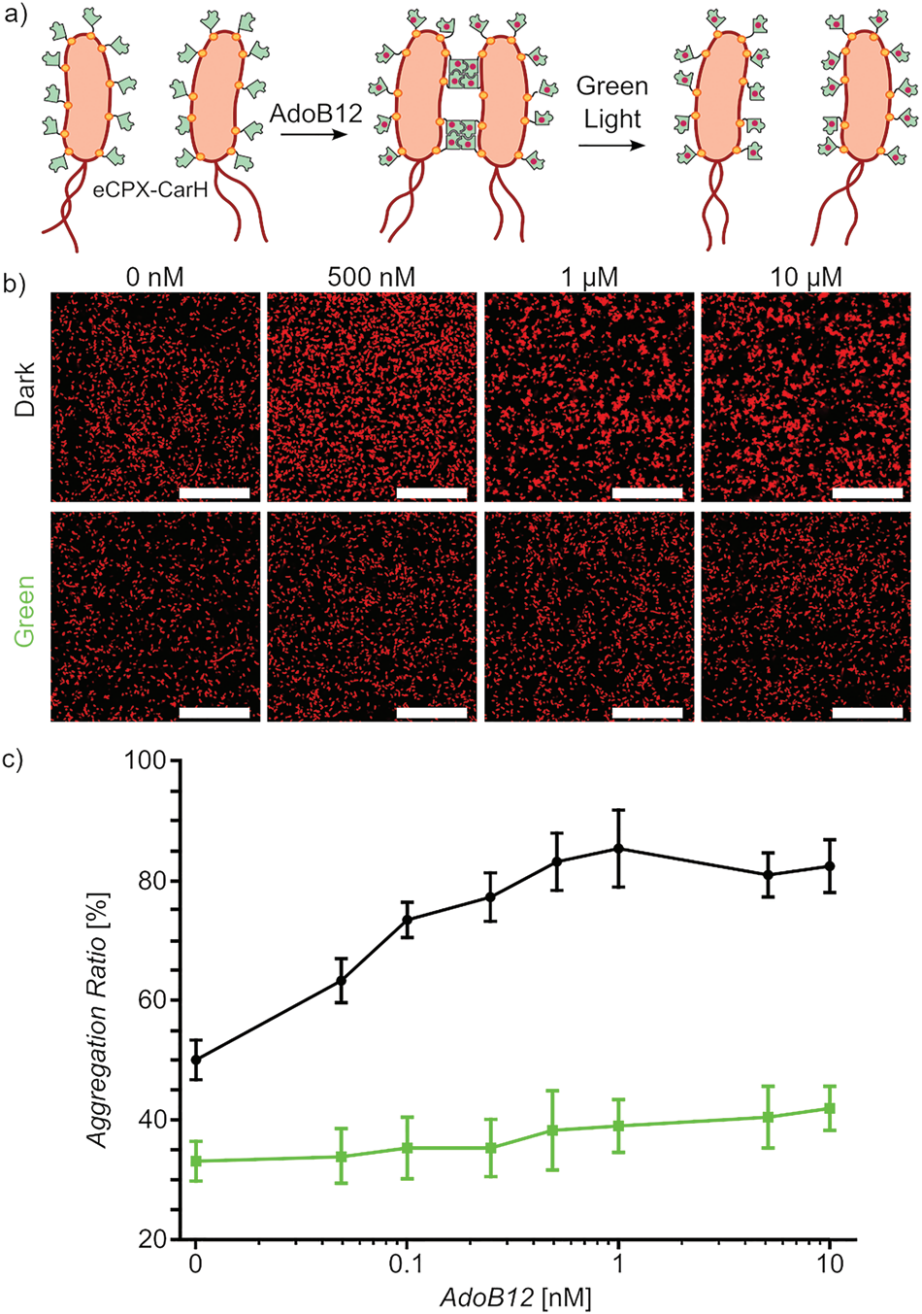
Design of AdoB12 whole‐cell biosensor based on CarH. a) Engineered bacteria that express CarH‐eCPX on their surfaces remain dispersed in the absence of AdoB12. When AdoB12 is added in the dark, bacteria aggregate due to the AdoB12 induced tetramerization of CarH. Under green light, the bacteria separate from each other as the CarH tetramer disassembles into its monomer. b) Confocal fluorescence microscopy images of *E. coli* MG1655 co‐expressing CarH‐eCPX and mCherry in the presence of AdoB12 in the dark and under green light. Scale bars are 50 µm. c) Aggregation ratio of bacteria in (b). The data is shown as the mean ±SD of three independent experiments.

For the design of the AdoB12 sensor, we fused the cobalamin‐binding domain (CBD) of CarH (referred to as CarH hereafter) to the N‐terminus of eCPX, a circularly permuted transmembrane carrier protein^[^ ^36^ ^]^ such that the CarH domain was displayed on the outer membrane of *E. coli* (**Figure** **1**a). A similar strategy has already been employed to successfully display other photoactive proteins at bacterial surfaces as adhesins.^[^ ^37^ ^]^ In the design, we expected surface available CarH‐eCP*X* to mediate bacterial adhesions in the presence of AdoB12 and promote bacterial aggregation as CarH forms a dimer‐of‐dimers type of tetramer that geometrically would require monomers from different bacterial surfaces to interact.^[^ ^28^ ^]^ Last, we also expected the bacterial adhesions to disassemble when illuminated with green light as the CarH tetramers dissociated into their monomers.

### 2.2 CarH Mediated Bacteria Cell–Cell Adhesions

In the first step, we aimed to validate that bacteria expressing CarH on their surfaces aggregate in the presence of AdoB12 in the dark and dissociate from each other when exposed to green light. For this purpose, *E. coli* MG1655 cells were cotransformed with CarH‐eCPX and mCherry plasmids and titrated with different concentrations of AdoB12 (0‐10 µм) in PBS (phosphate buffer saline). After 3 h incubation in the dark, bacterial aggregates were visible under a fluorescence microscope but not in samples incubated under green light illumination (Figure 1b).

The degree of bacterial aggregation depended on the concentration of AdoB12. As a measure of bacterial aggregation, the aggregation ratio was defined as the area occupied by clusters of bacteria divided by the total area occupied by all bacteria, and clusters of bacteria were defined as objects with an area >25 µm^2^, which corresponded to the size of ten single bacterial cells. We found that in the dark, even concentrations as low as 50 nм AdoB12 resulted in an increased aggregation ratio (Figure 1c). The aggregation ratio plateaued at concentrations above 500 nм. In comparison, the aggregation ratio remained equally low for samples kept under green light. This finding demonstrated that the aggregation was specifically due to the CarH tetramerization in the presence of AdoB12 in the dark as the aggregation was completely reversed under light illumination. In previous work, where CarH was expressed on the surface of mammalian cells, the highest extent of cell–cell adhesions was reached at 1 µм AdoB12 in the dark,^[^ ^35^ ^]^ which was similar to the 500 nм found here and also in accordance with the measured dissociation constant of 824 ± 240 nм measured for AdoB12 binding to CarH using isothermal titration calorimetry.^[^ ^27^ ^]^

### 2.3 AdoB12 Dependent Bacterial Agglutination

**Figure 2:**
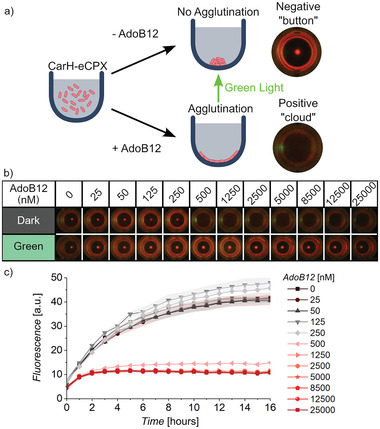
Agglutination assay for AdoB12. a) Design of the agglutination assay. Bacteria expressing CarH‐eCP*X* do not agglutinate in the absence of AdoB12 and settle to the bottom of the U‐bottom microtiter wells as visible buttons. In the dark in the presence of AdoB12, the bacteria agglutinate and form a thin layer in the well invisible to the naked eye. Under green light, the AdoB12 dependent agglutination reverses and a visible button forms even in the presence of AdoB12. b) Photographs of CarH‐eCP*X* and mCherry expressing bacteria incubated with different concentrations of AdoB12 in the dark and under green light after 16 h. c) mCherry fluorescence measured at the center of the well for samples in (b) over time. The data is shown as the mean ± SD of nine independent samples.

After this proof‐of‐concept, we implemented the AdoB12 sensitivity of the CarH‐mediated bacterial cell–cell adhesions into a bacterial agglutination assay. This assay would represent a simple, low cost, and specific AdoB12 biosensor with a visual readout that would not require expensive instruments such as microscopes.^[^ ^26^ ^]^ In the bacterial agglutination assay, 200 µL aliquots of the CarH‐eCPX bacteria cotransformed with a mCherry plasmid in PBS were placed into U‐bottom 96‐well microtiter plates with different concentrations of AdoB12 ranging from 0 nм to 25 µм and the bacteria were allowed to settle overnight at room temperature (**Figure** **2**a). In the absence or at low concentrations of AdoB12, the CarH‐eCPX bacteria precipitated to the bottom, visible to the naked eye as a button at the bottom of the wells. Above 500 nм of AboB12, the bacteria stuck together through the CarH mediated interactions and precipitated as a thin film/cloud that covers a wide area of the well (Figure 2b). The observed sensitivity of the sensor to 500 nм of AdoB12 is in agreement with the results from the bacterial aggregation as observed under the microscope. It should be noted that for analyses with concentrations above 500 nM, the concentration can be determined using serial dilutions and calculating the initial concentration using the dilution factor.

**Figure 3:**
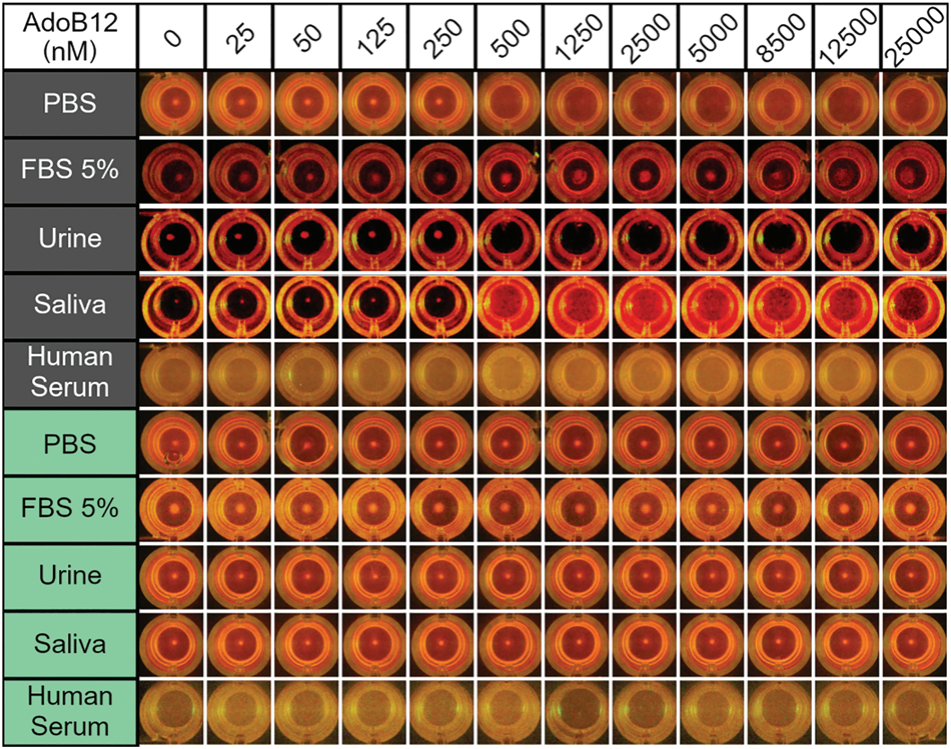
Specificity of the AdoB12 bacterial agglutination assay. a) Different vitamin B12 derivatives differ in their axial ligand. b) Fluorescence images of bacteria expressing CarH‐eCP*X* and mCherry incubated with various concentrations of AdoB12, MeB12, CNB12, and OHB12 in the dark.

Bacterial agglutination only occurred in samples incubated in the dark and control samples subjected to green light illumination for the entire duration of the assay did not agglutinate. The change in agglutination under green light demonstrates that the agglutination is due to the specific CarH interactions. The feature of samples that only agglutinate in the dark but not under green light can be used as an internal quality control to exclude false positive or false positives due to complex matrices or errors in the handling (see example later in **Figure** **3**). The initial handling of the CarH‐eCP*X* bacteria and setting up the assay under ambient laboratory light did not affect the assay performance as long as the samples were kept in the dark during the incubation step by wrapping the plates with aluminum foil or placing them in a closed incubator (Figure S1, Supporting Information). On the other hand, if the bacteria were kept under ambient light rather than green light, the bacteria didn't agglutinate. Thus, ambient light can be used instead of green light in the field.

Initially, we allowed the bacteria to settle overnight but in fact, the bacterial aggregation takes place already at earlier time points as observed under the microscope within 3 h (Figure 1b; Figure S2a and Movie S1, Supporting Information). As readout time is a key metric, we evaluated the performance of the agglutination assay over time and found that buttons in non‐agglutinated samples were already visible as early as 7 h (Figure S2b, Supporting Information). It is also possible to monitor bacterial agglutination using a readout other than the visual formation of the button at the bottom of the plate. By measuring the mCherry fluorescence of the bacteria at the center of the well, we found a faster increase in the mCherry fluorescence for samples with AdoB12 ≤ 250 nм than for samples at higher concentrations (Figure 2c). These differences were already detectable after 1 h and increased to a plateau within 8 h, as in non‐agglutinated samples, the bacteria concentrate more and faster at the center of the well compared to the agglutinated ones. The extra mCherry plasmid allows monitoring of the bacterial agglutination with better visual contrast and with an alternative readout but is not essential for and does not affect the bacterial agglutination assay. Bacteria only expressed CarH‐eCP*X* without the extra mCherry plasmid agglutinated in the presence of AdoB12 with the same sensitivity; however, were more difficult to photograph (Figure S3, Supporting Information). Bacteria only expressing mCherry but no CarH showed no agglutination under the same conditions. The use of a cell‐based sensor may lead to concerns in terms of shipping of the sensor and deployment in low‐tech settings. To overcome these hurdles, we lyophilized the CarH‐eCP*X* bacteria and evaluated the sensor performance after rehydration in PBS. We found that after the freeze‐drying, the sensing capabilities remained unaffected (Figure S4, Supporting Informatio). The expression of CarH‐eCP*X* was under the control of an arabinose‐activated promoter, which allowed altering of the expression levels of CarH on the bacterial surface. We found that less than 0.02% arabinose used in all other assays resulted in a lack of AdoB12 response but higher concentrations did not affect the sensitivity (Figure S5, Supporting Information). Taken together, the clear visual readout of the agglutination assay depending on the AdoB12 concentration makes this low‐cost technique suitable for many budget‐constrained applications as it only requires a photographic device, which is much cheaper than any equipment previously discussed.^[^ ^12^, ^13^, ^14^, ^15^, ^16^, ^17^, ^18^ ^]^

### 2.4 Specificity for AdoB12 over Other Vitamin B12 Derivatives

**Figure 4:**
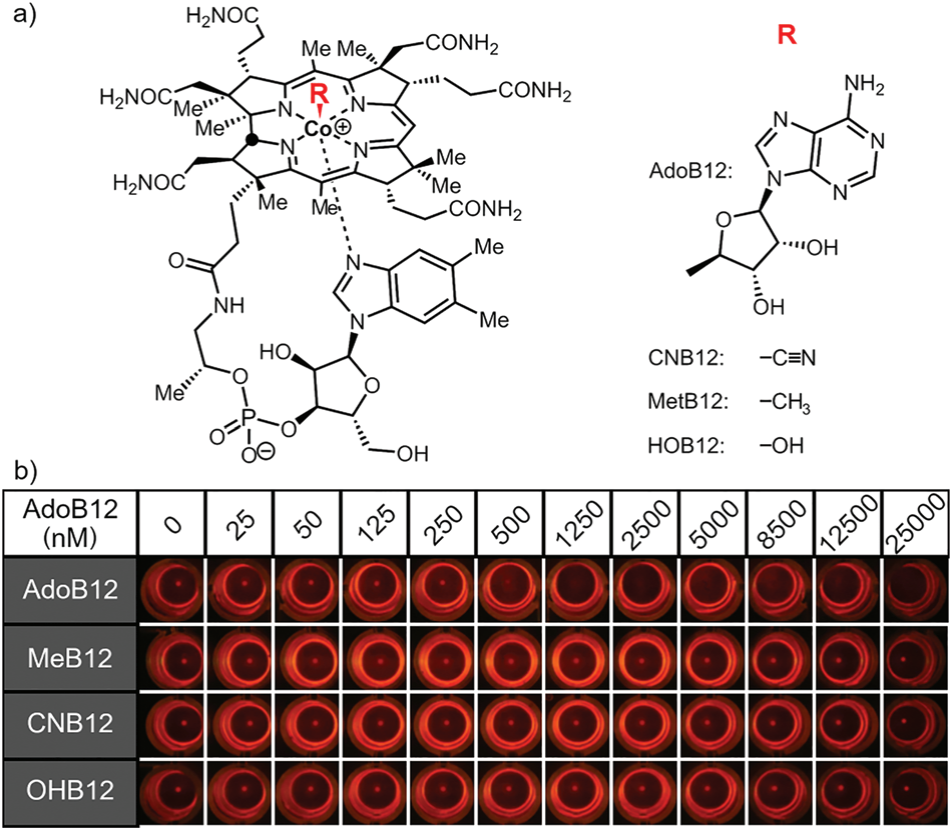
Performance of the AdoB12 bacterial agglutination assay with different vitamin B12 supplements in the dark. Brands 1 and 2 have AdoB12, Brand 3 has a 1:4 mixture of AdoB12:MeB12, Brand 4 has MeB12, and Brands 5 and 6 have CNB12.

Next, we evaluated the specificity of CarH‐eCP*X* toward AdoB12 over other types of cobalamin. Therefore, we repeated the agglutination assay with MeB12 as the other bioactive form of vitamin B12, CNB12 as an industrially produced form commonly used in food supplements and OHB12 as a hydrolysis product (**Figure** **4**a). We found bacteria agglutination only in the presence of AdoB12 above 500 nм and no agglutination in the presence of any of the other forms of cobalamin (Figure 4b). Further, control samples kept under green light illumination did not agglutinate (Figure S5, Supporting Information). This high specificity was a result of the preference of CarH to bind to AdoB12 and the fact that the sensor responds to the extracellular pool of vitamin B12. It is known that cells have the ability to convert MeB12, CNB12, and OHB12 into AdoB12 through specific enzymes that replace the axial ligands on the cobalamin.^[^ ^10^, ^38^ ^]^ Yet, as the CarH‐eCP*X* operates on the exterior of the bacterial cells, the intracellular conversion of different forms of vitamin B12 into AdoB12 doesn't seem to affect performance and allows it to operate as an AdoB12‐specific biosensor. In addition, the extracellular detection of AdoB12 is also an advantage as it circumvents issues that arise from the transport into the cell, which has been recognized as a bottleneck for vitamin B12 biosensors.^[^ ^20^ ^]^

### 2.5 Applications of the AdoB12 Sensor

**Figure 5:**
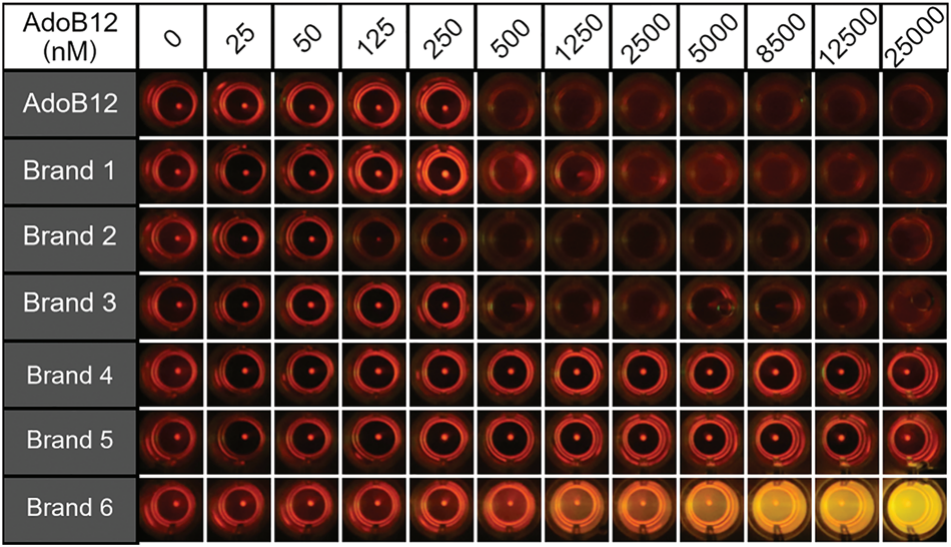
Performance of the AdoB12 bacterial agglutination assay with vitamin B12 in different biological fluids in the dark. The AdoB12 sensor performs correctly in urine and saliva matrix but not in protein‐rich serum matrices.

Next, we employed the AdoB12 biosensor to analyze different commercially available vitamin B12 supplements. While less expensive and common supplements usually contain CNB12, AdoB12 and MeB12‐containing supplements advertise to already contain the bioactive forms and are available at high doses (1000 µg per day) without prescription. In this study, we tested supplements with pure AdoB12 (Brand 1, liquid and Brand 2, solid), mixtures of AdoB12 and MeB12 (Brand 3, 1:4 ratio, liquid), pure MeB12 (Brand 4, liquid), only CNB12 (Brand 5, solid) and CNB12 in mixture with other vitamins (Brand 6, solid). Samples in pill‐form were crushed, suspended in water, and filtered. For all brands, the cobalamin concentration was estimated using the absorbance band at 550 nm and the agglutination assay was performed as before (**Figure** **5**). In the dark, all AdoB12 supplements (Brands 1–3) with concentrations >500 nм showed agglutination and no pellets were visible by eye, while wells with other cobalamin sources (Brands 4–6) did not agglutinate and precipitated to the bottom of the wells. In the control samples that were incubated under green light, no agglutination was overserved for brands whatsoever, which demonstrates the specific response to AdoB12 even in various matrices (Figure S7, Supporting Information).

Finally, we investigated if the AdoB12 sensor performed in various biological fluids that may be of diagnostic relevance. To this end, we performed the agglutination assay in the presence of fetal bovine serum (FBS), urine diluent, artificial saliva, and human serum (Figure 3). In FBS, the sensing was impaired as the bacteria visibly proliferated and formed buttons at all AdoB12 concentrations (false negative). Likewise, for tests in human serum, results were inconclusive as no pellet formed even at low AdoB12 concentration (false positive). In these cases, the high protein content in serum seemingly impaired the AdoB12 induced agglutination. On the other hand, we found that the sensor detected AdoB12 with the same sensitivity of 500 nм in urine and saliva matrix. The absence of proteins in these two synthetic matrices is probably important for the good performance of the sensor. In urine of healthy individuals, protein concentrations are low and we expect similar performance of the sensor. Yet, in complete saliva, the protein concentration is high and the impact of this needs to be investigated further for saliva‐based diagnostics. The specificity of the response in urine and saliva was further confirmed in experiments under green light, where the agglutination was reversed. In contrast, the agglutination didn't change under green light for samples in FBS and human serum, which gave false negative and false positive results, respectively. In these cases, the lack of light response allowed us to identify the interference by the matrix.

Measurements of protein complexed cobalamin and bioactive cobalamin in serum were used to monitor vitamin B12 status using blood tests.^[^ ^2^ ^]^ Urine tests that measure vitamin B12 status through the metabolite methylmalonic acid represent a noninvasive alternative but still require a laboratory setting and detect vitamin B12 indirectly. The here presented AdoB12 sensor could complement the urine‐based testing possibilities. In particular, patients who receive mega doses of cobalamin of 1000 µg per day and above^[^ ^8^ ^]^ exceed the absorption limit of a maximum of 40 µg per day^[^ ^4^, ^39^ ^]^ and excrete the excess through the urine. Mollin and Ross et al. have found ≈500 µg vitamin B12 in the urine within 24 h after 1000 µg intramuscular injection.^[^ ^40^ ^]^ Assuming normal urine output, this would correspond to a range of ≈160–600 nm vitamin B12, which was within the sensitivity range of the here described sensor. The selectivity for AdoB12 may be of particular relevance for patients with hereditary vitamin B12 deficiency that have defects in genes for AdoB12 synthesis (*cblA* or *cblB*)^[^ ^11^ ^]^ and must supplement particularly this form of vitamin B12 at high doses. In addition, many people take high doses of AdoB12 supplements, which are available over the counter. In both cases, the here presented AdoB12 sensor used in urine samples could be a preferred alternative for monitoring the therapy/supplementation over current blood‐based methods.

## 3 Conclusion

Here, we demonstrated the proof‐of‐concept for an AdoB12 sensitive whole‐cell biosensor that builds on bacterial agglutination. In this biosensor, the bacteria express the chimeric protein CarH‐eCPX on their surface. The CarH displayed on the bacterial surface retains its ability to form tetramers in the presence of AdoB12, which leads to the aggregation of the bacteria. The fact that the agglutination is reversible under green light shows that the bacterial cell–cell adhesions are specifically mediated by CarH tetramers that dissociate into their monomers under green light illumination and can serve as an internal control. The agglutination assay further shows excellent specificity toward AdoB12 over other forms of vitamin B12, which results from the binding preference of CarH as is also demonstrated with commercially available vitamin supplements. Moreover, the agglutination assay works in urine equally well, which opens the possibility to monitor excess AdoB12 secretion when taking high‐dose supplements. The agglutination assay for AdoB12 has a straightforward visual readout without the need for specialized equipment or trained personnel, which makes it practical and low‐cost to implement at the point‐of‐care.

## 4 Experimental Section

### Strains and Plasmids

The plasmid pBAD33.1, which has a chloramphenicol selectable marker and an l‐arabinose inducible promoter, was a gift from Christian Raetz (Addgene plasmid #36267).^[^ ^41^ ^]^ The sequence coding for the cobalamin binding domain of CarH fused at its C‐terminus to the N‐terminus of eCPX, was synthesized by GeneScript and cloned downstream to the araBAD promoter in pBAD33.1 to result in the CarH‐eCPX plasmid. The CarH‐eCPX plasmid was transformed into *E. coli* K‐12 MG1655. Single colonies of bacteria were co‐transformed with the CarH‐eCPX plasmid and pTRC99a‐mCherry (ampicillin selectable marker, a gift from Prof. Victor Sourjik, Max Planck Institute for Terrestrial Microbiology) was inoculated in 10 ml LB media supplemented with chloramphenicol (35 µg mL^−1^), ampicillin (50 µg mL^−1^), and l‐arabinose (0.02%) and grown overnight at 37 °C while shaking at 150 rpm.

### Other Materials

Pure cobalamins (AdoB12, MeB12, CNB12, and HOB12), artificial saliva (Cat# SAE0149), urine diluent (Cat# SAE0074), FBS, and all other chemicals were purchased from Sigma–Aldrich. TruLab N human serum was acquired from DiaSys. Different food supplements with vitamin B12 were acquired from different suppliers (ZellKraft, NCM, Aportha, Vitamaze, Test‐sept, and Vitaverlan).

### Bacterial Aggregation

Overnight cultures of *E.coli* transformed with CarH‐eCPX and pTRC99a‐mCherry were harvested by centrifugation, washed with PBS, and then, diluted to an OD600 of 0.1 (≈1 × 10^8^ cells mL^−1^). Aliquots of 300 µL bacterial suspension were added into 8 well µ‐slide chambers (ibidi) alongside 0–10 µм AdoB12. The sample was statically incubated for 3 h at room temperature in the dark (in an opaque container or fully wrapping the plate in aluminum foil) or green light illumination (520 nm LED, 50 l×). An area of 2 × 2 mm in the center of the wells was imaged using a confocal fluorescence microscope (Leica DMi8, 552 nm laser, 40× objective). Later, the images were analyzed with ImageJ particle analyzer;^[^ ^42^ ^]^ the aggregation ratio was defined as the area occupied by bacterial clusters (objects with an area > 25 µm^2^, corresponding to the size of around ten single bacteria) divided by the total area occupied by all bacteria. Experiments were performed in three technical replicates with three biological replicates each time.

### Bacterial Agglutination

The agglutination assay was adapted from previously described protocols.^[^ ^26^ ^]^ In short, 200 µL of bacteria suspension at an OD600 of 0.1 in PBS was incubated with 0–25 µм AdoB12 or other derivatives in a clear round‐bottom 96‐well plate (Greiner Bio One). The plates were statically incubated in the dark or green light illumination (520 nm LED, 50 l×) overnight (≈16 h) at room temperature. The fluorescence of mCherry was measured at the center of the well hourly using a plate reader (TECAN Spark) (excitation 545 ± 10 nm, emission 600 ± 25 nm). Photographs of the assay were taken on a Nippon FastGene FAS‐DIGI PRO imaging system with blue‐green LED illumination and an amber FAS‐Nano Amber Filter shield in front of the camera lens.

### Statistical Analysis

Confocal microscopy images were converted into binary images for analysis with automatic global thresholding using the IJ_IsoData algorithm in ImageJ. All data was displayed as arithmetic mean ± SD. All experiments were at least performed as independent triplicates.

## Conflict of Interest

The authors declare no conflict of interest.

## Supporting information

Supporting Information

Supplemental Movie 1

## Data Availability

The data that support the findings of this study are available from the corresponding author upon reasonable request.
