# Supplementary material for: An Adenosylcobalamin Specific Whole‐Cell Biosensor
Source: Adv Healthc Mater. 2023 May 1;12(25):2300835. doi: 10.1002/adhm.202300835 (PMC11468855; doi:10.1002/adhm.202300835)
Supplement: Supplementary file 1 — Supporting Information [file ADHM-12-2300835-s002.pdf]

ADVANCED HEALTHCARE MATERIALS

Supporting Information

for *Adv. Healthcare Mater.*, DOI 10.1002/adhm.202300835

An Adenosylcobalamin Specific Whole-Cell Biosensor

*Juan José Quispe Haro and Seraphine V. Wegner**

WILEY-VCH

Supporting Information

An Adenosylcobalamin Specific Whole-Cell Biosensor

*Juan José Quispe Haro and Seraphine V. Wegner**

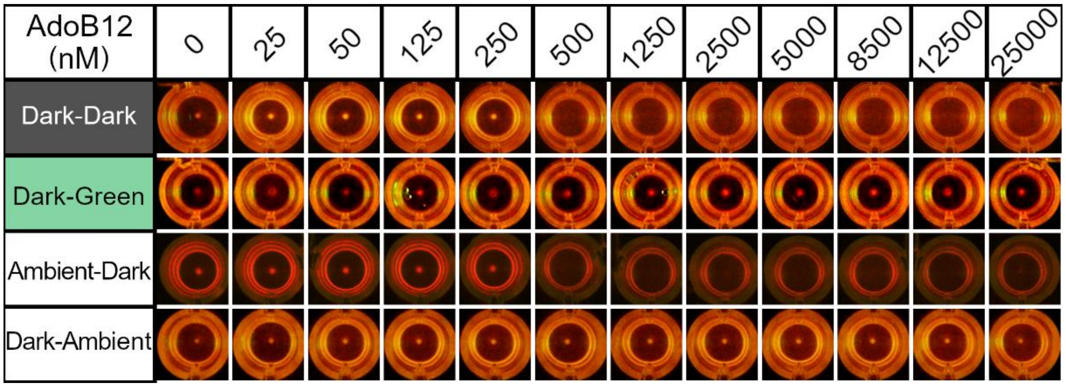

Figure S1. Performance of the AdoB12 agglutination assay prepared under different conditions. CarH-eCPX and mCherry expressing bacteria were pipetted into the U-bottom wells in the dark or under ambient light (first label) and subsequently incubated for 16 h either in the dark, under green light or ambient light (second label). Setting up the agglutination assay in the dark or under ambient light did not alter the performance of the assay. Only the longtime incubation under green or ambient light stopped the agglutination at higher concentrations of Ado B12.

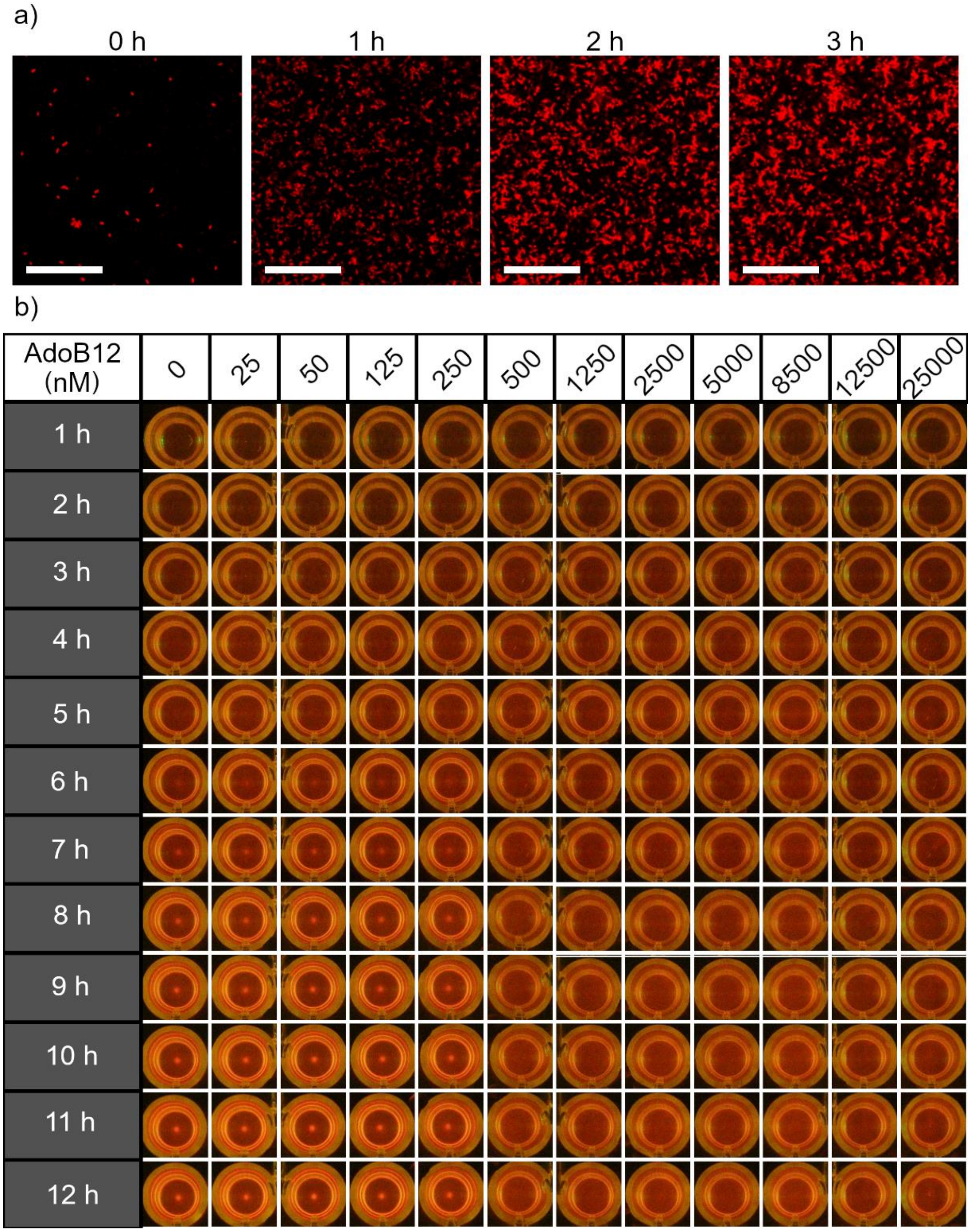

Figure S2. Performance of the AdoB12 agglutination assay over time in the dark. (a) Time frames of CarH-eCPX and mCherry expressing bacteria aggregating in presence of 1000 nM AdoB12. Scale bars are 50 µm. (b) Photographs of the agglutination assay with bacteria expressing CarH-eCPX and mCherry at different time points. Buttons were visible as early as 7 h after setting up the assay at concentrations below 250 nM.

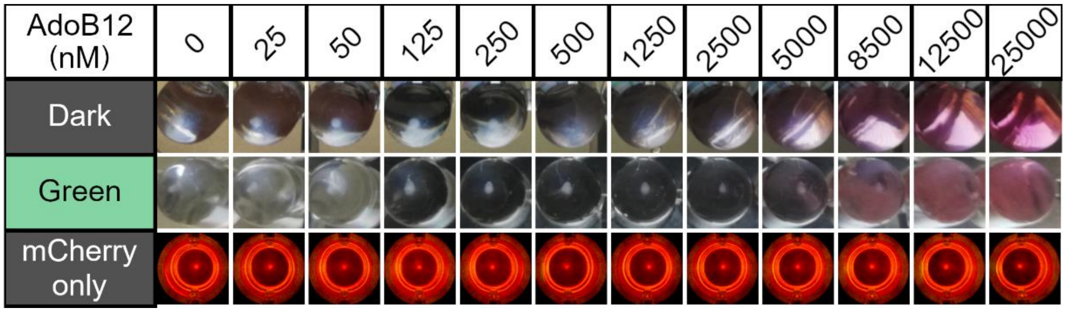

Figure S3. Photographs of the agglutination assay for AdoB12 with bacteria expressing CarH-eCPX without mCherry label incubated with various concentrations of AdoB12 in the dark and under green light. At AboB12 concentrations above 500 nM in the dark, the button at the bottom of the well was no longer visible. In samples incubated under green light, the button was visible at all concentrations of AdoB12. Bacteria expressing only mCherry show no agglutination and buttons at all AdoB12 concentrations tested.

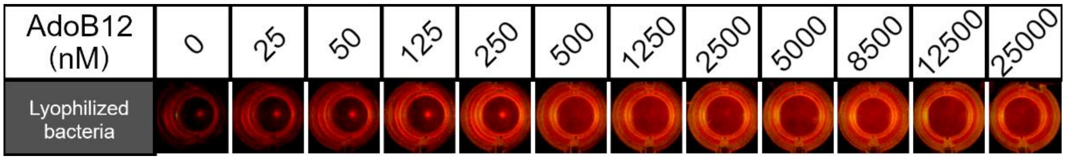

Figure S4. Agglutination assay for AdoB12 with CarH-eCPX and mCherry expressing bacteria that were lyophilized and later resuspended in PBS. The performance of the sensor was the same as the original sensor.

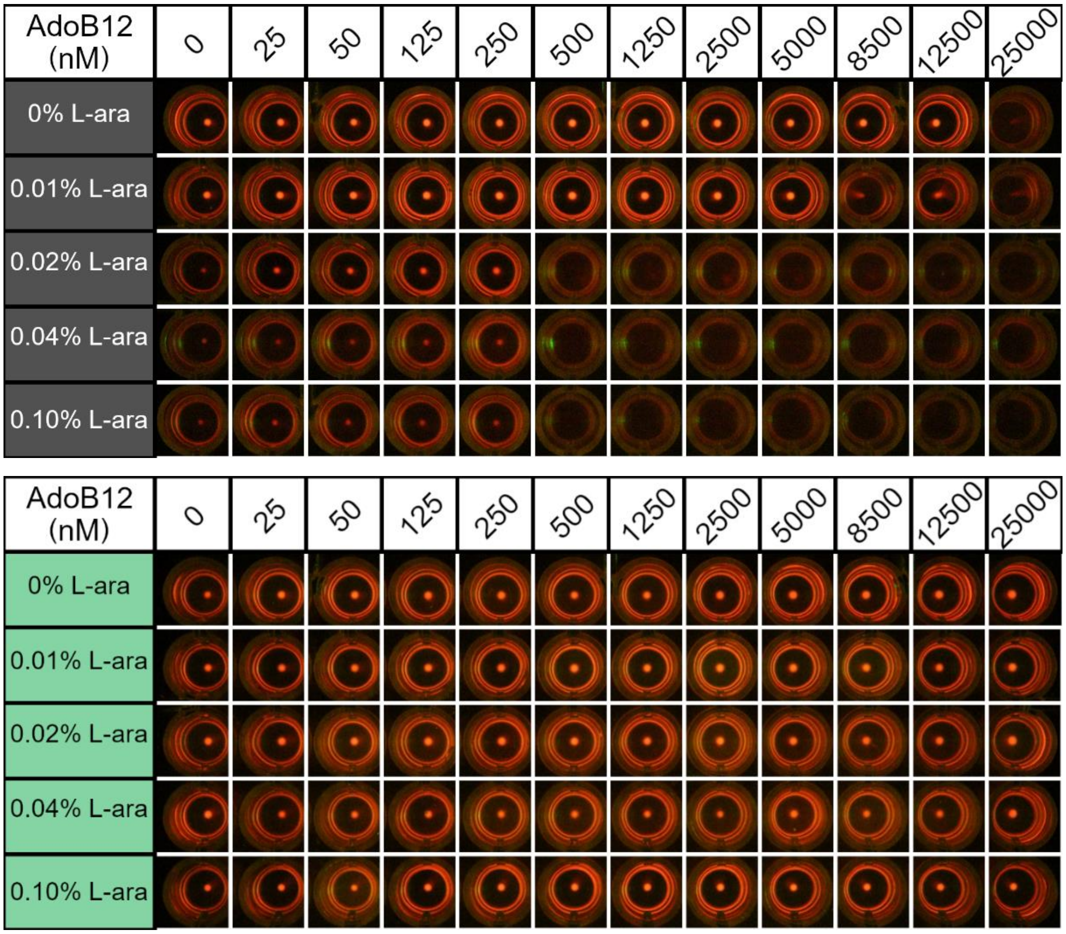

Figure S5. Agglutination assay for AdoB12 with bacteria expressing CarH-eCPX at different levels. Fluorescence images of bacteria expressing CarH-eCPX and mCherry incubated with various concentrations of *L*–arabinose and AdoB12 in the dark and under green light. The expression of surface available CarH-eCPX is inducible with *L*–arabinose. In absence of the inducer, CarH-eCPX is not produced by the bacteria and thus no agglutination was observed in presence of AdoB12. At low *L*-arabinose concentrations (0.01%), the agglutination was still not observable in the presence of AdoB12 as the CarH-eCPX expression was too low. At concentrations above 0.02% *L*-arabinose, the bacteria agglutinated at AdoB12 concentrations above 500 nM in the dark, which was reversible under green light illumination. 0.02% *L*-arabinose was used in all other experiments.

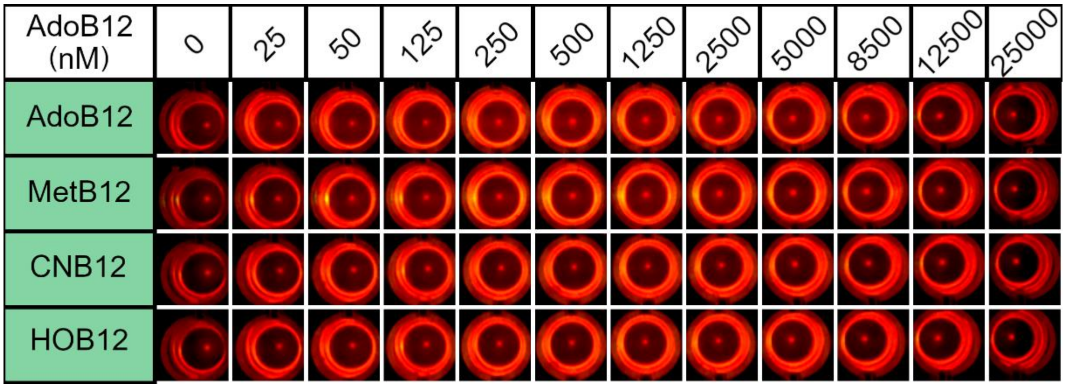

Figure S6. Agglutination assay for different vitamin B12 derivatives under green light. Fluorescence images of bacteria expressing CarH-eCPX and mCherry incubated with various concentrations of AdoB12, MeB12, CNB12 and OHB12 under green. Negative control experiment complementary to Figure 3, showed that agglutination does not occur for any vitamin B12 derivatives under green light illumination.

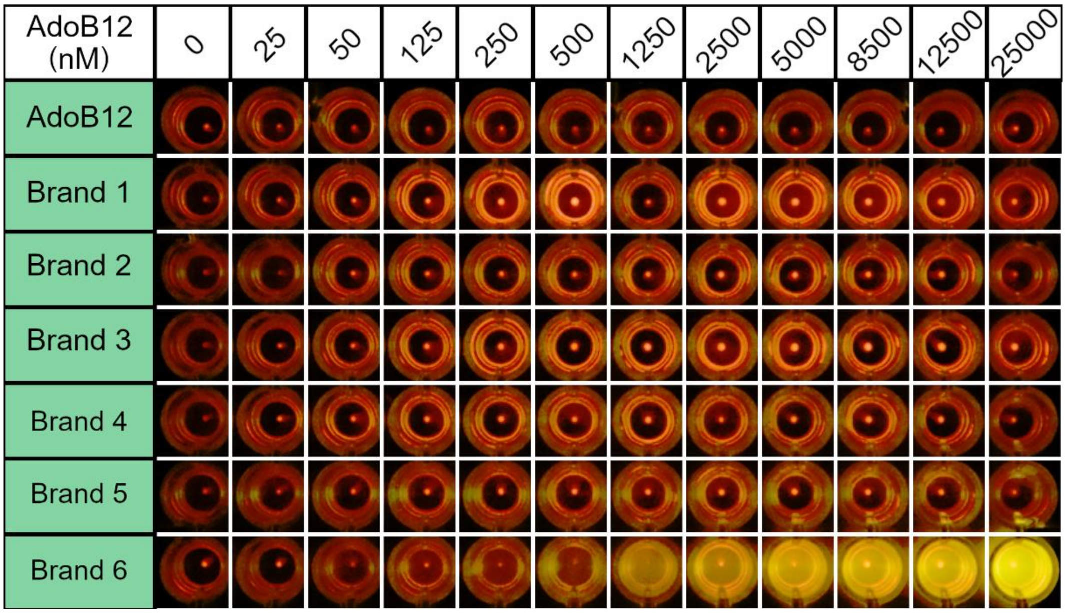

Figure S7. Performance of the AdoB12 bacterial agglutination assay with different vitamin B12 supplements under green light. Brand 1 & 2 contain have AdoB12, Brand 3 has a 1:4 mixture of AdoB12:MeB12, Brand 4 has MeB12, Brand 5 & 6 have CNB12. Negative control experiment complementary to Figure 4, showed that agglutination does not occur for any vitamin B12 supplements under green light illumination.
